# Metals and kidney markers in adult offspring of endemic nephropathy patients and controls: a two-year follow-up study

**DOI:** 10.1186/1476-069X-7-11

**Published:** 2008-04-03

**Authors:** Wilfried Karmaus, Plamen Dimitrov, Valeri Simeonov, Svetla Tsolova, Angel Bonev, Rossitza Georgieva

**Affiliations:** 1 Department of Epidemiology and Biostatistics, University of South Carolina, Columbia, South Carolina 29208, USA; 2 National Center of Public Health Protection, Sofia, Bulgaria; 3 Vratza District Hospital, Vratza, Bulgaria

## Abstract

**Background:**

The etiology of Balkan Endemic Nephropathy, (BEN), a tubulointerstitial kidney disease, is unknown. Although this disease is endemic in rural areas of Bosnia, Bulgaria, Croatia, Romania, and Serbia, similar manifestations are reported to occur in other regions, for instance Tunisia and Sri Lanka. A number of explanations have been stated including lignites, aristolochic acid, ochratoxin A, metals, and metalloids. Etiologic claims are often based on one or a few studies without sound scientific evidence. In this systematic study, we tested whether exposures to metals (cadmium and lead) and metalloids (arsenic and selenium) are related to Balkan Endemic Nephropathy.

**Methods:**

In 2003/04 we recruited 102 adults whose parents had BEN and who resided in one of three communities (Vratza, Bistretz, or Beli Izvor, Bulgaria). A control group comprised of 99 adults having non-BEN hospitalized parents was enrolled in the study during the same time. We conducted face-to-face interviews, ultrasound kidney measurements, and determined kidney function in two consecutive investigations (2003/04 and 2004/05). Metals and metalloids were measured in urine and blood samples. To assess the agreement between these consecutive measurements, we calculated intraclass correlation coefficients. Repeated measurement data were analyzed using mixed models.

**Results:**

We found that cadmium and arsenic were associated with neither kidney size nor function. Lead had a significant but negligible effect on creatinine clearance. Selenium showed a weak but significant negative association with two of the four kidney parameters, namely creatinine clearance and β_2_-microglobulin. It was positively related to kidney length. These associations were not restricted to the offspring of BEN patients. Adding credence to these findings are reports showing comparable kidney effects in animals exposed to selenium.

**Conclusion:**

The findings of this 2-year follow-up study indicate that metals and metalloids do not play a role in the etiology of Balkan Endemic Nephropathy. Against the assumption in the literature, selenium was not protective but a risk factor. Since comparable associations were observed in animals, future studies are needed to explore whether selenium may have adverse renal effects in humans.

## Background

Balkan Endemic Nephropathy (BEN) is a tubulointerstitial kidney disease that progresses slowly over many years. First characterized in the Vratza District, Bulgaria in 1956 [1], similar nephropathies were subsequently described in Yugoslavia [2,3] and in Romania [4]. In 1964 this disease was recognized as a new nosological entity and named Balkan Endemic Nephropathy. The final disease stage is characterized by renal failure and shrinkage of both kidneys to the size of walnuts [5]. BEN shows familial clustering and develops only in certain areas in the Balkan countries – Bulgaria, Romania, Serbia, Montenegro, Croatia, and Bosnia and Herzegovina. Its spatial distribution shows a mosaic-like pattern, with some villages afflicted for decades, whereas others situated in the same vicinity have remained free of BEN [6-8]. It is still unknown what causes BEN. Although this disease is endemic in rural areas of the Balkan, endemic nephropathies are reported to occur in other regions. For instance, chronic kidney diseases of uncertain etiology, which resemble BEN, have been reported in Sri Lanka (personal communication, Dr. Alturaliya, Sri Lanka). Every now and then, new explanations emerge emphasizing lignites [9], aristolochic acid [10], ochratoxin A [11], metals, and metalloids [12-15]. However, these claims are often not substantiated by sufficient evidence or scientific studies. For instance, studies claim that exposures to metals (cadmium and lead) and metalloids (arsenic and selenium) are related to the disease, with selenium being protective [12-15].

An important characteristic of BEN is the late onset of the disease (not before age 50) and thus probably a long latency period and/or a period of being at risk between exposure and the gradually developing clinical manifestation of the disease. To determine exposures before the onset of the disease, we chose to study the offspring of patients with BEN, who are at a higher risk for the disease, and use the offspring of other non-BEN patients as a control group.

Early markers for BEN are a lower creatinine clearance, increased excretion in urine of total protein, albumin, and β_2_-microglobulin, and symmetrically decreased kidney measures [16-27]. We recently reported in a cross-sectional investigation that changes in these markers are associated with being offspring of BEN parents [28]. In this work we link clinical markers with the concurrent burden of metals and metalloids.

We investigated four metals and metalloids: lead, arsenic, cadmium, and selenium. Lead, absorbed in blood, is bound extensively to erythrocytes (90–95%) and lead content in whole blood is used routinely as a biomarker of exposure (up to a few months before sampling) [29,30]. Most arsenic absorbed by the lungs or by the gastro-intestinal tract is excreted in the urine, mainly within 1–2 days. Measurement of urinary arsenic levels is generally accepted as the most reliable indicator of recent exposure [31]. Total arsenic in urine, which is measured in this study, includes inorganic arsenic, as well as its metabolites monomethylarsonic acid and dimethylarsinic acid.

Cadmium is a heavy metal which accumulates predominantly in the kidneys (half-life: 15–40 years) and liver and is excreted mainly in urine. In low-level exposures (below 10 μg/g creatinine) cadmium content in urine reflects body burden and is used as a biomarker of cumulative exposure [30,32]. After absorption, selenium in blood is distributed between red blood cells and plasma/serum. The concentration in plasma or serum is considered to reflect short-term exposure, whereas the selenium content of erythrocytes indicates long-term exposure [32]. In a steady state, the Se levels in red blood cells are considered to be higher than those in serum [33,34], however, both are significantly correlated and comparably associated with daily intake [35-37]. Serum selenium is used for assessment of occupational and environmental selenium exposure, including nutritional status, in various studies, for instance, the U.S. National Health and Nutrition Examination Survey [32,38,39].

Based on prior reports in the BEN literature we formulated five hypotheses:

1) Selenium serum concentration is protective against adverse kidney effects.

2) Lead in blood, 3) cadmium in urine, and 4) arsenic in urine are associated with adverse alterations in BEN kidney markers and size.

5) Adult offspring with a parental history of BEN show stronger associations between metals and/or arsenic and kidney variables.

**Figure 1:**
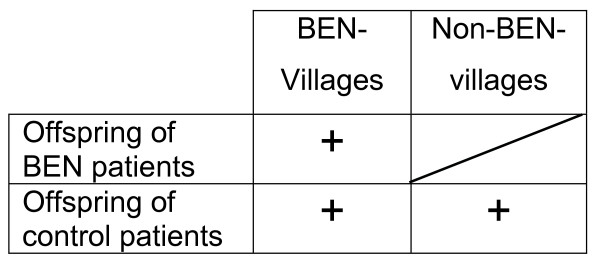
Study designs for Balkan Endemic Nephropathy.

Since the incidence of BEN shows a spatial distribution with a mosaic-like pattern one may consider to simultaneously compare residents from BEN villages and non-BEN villages and specific exposures. By doing so, we could compare combined effects of residence and of specific exposures. This would be advantageous, since we would also control for unknown risk factors acting in BEN villages. However, we need to take into consideration that we do not have BEN patients from non-BEN villages (Figure 1). Thus, a formal testing of interaction terms will not work. Alternately, if we compare the effect of metals in BEN-offspring from BEN villages with the effect of metals in non-BEN offspring from non-BEN villages, we would mix two effects: the specific metal effect and an unknown spatial effect, which would result in confounding. To estimate spatial effects, ecological studies have been conducted and have attributed differences to specific factors [14]. We considered that testing of specific risk factors requires a more rigorous comparison, namely of offspring of BEN and non-BEN patients from BEN villages.

## Methods

### Study Population

From October, 2003, to April, 2004, we recruited 102 adults whose father and/or mother were registered in the Vratza Hospital as BEN patients and who resided in one of three communities (Vratza, Bistretz, or Beli Izvor, Bulgaria). The parental diagnosis of BEN was based on epidemiological, clinical/laboratory, and pathological/anatomical criteria [40]. Regarding epidemiology, the region is known to have a high prevalence of BEN. The individual criterion is onset after the age of 50. Clinical and laboratory criteria are as follows: obscure onset, chronic course, absence of edema, and normochromic anemia in the more advanced phases. Pathologically, an almost symmetrical macroscopic shrinkage of the two kidneys is found. A control group of nearly equal size, 99 adult offspring of non-BEN hospitalized patients was enrolled at the same time. Diagnoses in control parents included diabetes mellitus, cardiovascular disorders, and liver problems. Only three of the 99 control subjects had parents with kidney diseases (one paternal kidney cancer not related to BEN and two maternal pyelonephritis cases). Subjects of both groups were frequency-matched according to gender and ten-year age groups. The population was enrolled and examined in 2003/04 and re-examined in 2004/05, which provided us with two repeated measurements one year apart.

All participants provided written consent through a procedure approved by the Institutional Review Board (human subject research committee) of the National Center of Public Health Protection, Sofia, Bulgaria. This research was reviewed and approved by the Commission of Medical Ethics at the National Center of Hygiene, Medical Ecology and Nutrition, Sofia, Bulgaria, on December 16, 2002.

### Interviews

We conducted face-to-face interviews with all participants, either in the hospital or by visiting them in their home villages. The standardized questionnaire asked for the place of residence, type of water supply, diet, smoking and drinking habits, medical symptoms, family history of BEN, family history of other kidney diseases and kidney tumors, and occupational history.

### Physical examination and determination of kidney sizes

Physical examinations, performed by an experienced physician with board certification in internal diseases and nephrology, assessed the general health status of the study subjects, including height and weight, and looked for symptoms of BEN and/or other internal diseases. Blood pressure was measured according to standards set by the World Health Organization [41].

Ultrasound examination of both kidneys included the patient lying on the left side and then on the right side, and took 20–30 minutes. The longest dimension of the kidney, the thickness of the kidney parenchyma (in the thinnest or minimal part), and the relationship of parenchyma and pyelon were measured. Data gathered also included parenchyma structure and the location, size, and morphology (cysts, stones, and tumors) of the kidneys. The images were saved electronically for future reference. The ultrasonographer (AGB) worked in the Department of Image Diagnostics, Vratza District Hospital, and was blinded to the clinical status of the participant (BEN or control offspring).

### Urine and blood collection and determination of kidney markers

Prior to the examination day, study subjects were provided with 100 mL polypropylene containers and instructed to collect their urine, avoiding external contamination, immediately after rising in the morning and to take it to the laboratory without delay. They were also told to refrain from eating fish, seafood, chicken, and organ meat during the week before sample collection in order to diminish their intake of organic arsenic via diet. At the laboratory, aliquots of the first morning urine sample were transferred to polypropylene tubes: one aliquot was used for the measurement of creatinine; the other two were acidified with either 1N HCl (Merck) for arsenic determination or 1N HNO_3 _(Merck) for cadmium determination. Then the aliquots were frozen at -20°C.

After the morning urine collection, the participants underwent a 4-hour examination with urine and blood sampling. Study subjects were asked to empty their bladders and were given 0.5 L of water to drink. From that moment on, all their urine was collected in polyethylene containers and refrigerated: At the end of the 1^st ^hour and at 4 hours the study subjects were asked to urinate. Venous blood was collected after 2 hours.

The hour-one urine was checked with pH-indicator paper strips (Merck). If the pH was lower than 7, a portion of the urine was alkalized by adding 1N NaOH until a pH equal to 7 or between 7 and 8 was reached. Creatinine and β_2_-microglobulin were measured in aliquots of the hour-one urine sample. The volume of the hour-one urine sample was taken into account when the total urine volume was recorded at the end of the 4-hour period. The total 4-hour urine sample was apportioned into aliquots and frozen at -20°C. As described elsewhere [28], we determined total protein, albumin, and creatinine in urine in the 4-hour sample. To adjust for creatinine concentration, it was measured three times: in the first morning, the hour-one, and the 4-hour samples.

Venous blood for hematology, the determination of lead, and for serum separation was taken in the middle of the 4-hour urine collection period. Blood samples for determination of lead were drawn in K_2_EDTA vacutainers. Serum was separated for the analyses of selenium and creatinine.

### Creatinine clearance

Creatinine clearance (CCR) was estimated from serum creatinine values by using the Cockcroft and Gault (1976) formula [42]. Specifically,

$$\text{CCR }(\text{ml}/\min)=\frac{(140-\text{age})\times \text{body weight in kg}}{\left(\text{serum creatinine}\times 72\right)}(\times 0.85\;\text{for females})$$

We adjusted CCR by dividing through body surface area [= square root ((height in cm × weight in kg)/3600)].

### Analyses of trace elements

Lead, cadmium, arsenic, and selenium were determined using Perkin-Elmer atomic absorption spectrometers (Bodenseewerk Perkin-Elmer, Ueberlingen, Germany). A Model 4110 ZL atomic absorption spectrometer (AAS) with a transverse heated graphite atomizer (THGA) and longitudinal Zeeman-effect background correction equipped with an AS-72 autosampler, electrodeless discharge lamps (EDL) System II, software WinLab (Version 1.2) and both "Standard" (Part No B 300–0643) and "End-capped" (Part No B 300–0644) THGA tubes with integrated platforms was used for direct electro-thermal AAS measurements of lead in blood, selenium in serum, and cadmium in urine. A Model 3030 AAS with a Mercury/Hydride System MHS-20, automatic deuterium background corrector and EDL System II was used for direct hydride generation AAS determinations of arsenic (As) in urine.

To measure lead (Pb), blood was diluted 10-fold with 0.25% v/v Triton X-100; 10 μL of the sample and 25 μg of (NH_4_)_2_HPO_4 _as a chemical modifier were injected into the THGA, pre-treated with 250 μg of zirconium and 20 μg of iridium [43]. For selenium (Se), serum was diluted 5-fold with 0.1% v/v Triton X-100 in 0.2% HNO_3_; 10 μL injections of the sample and 10 μg of rhodium modifier were injected into the THGA; standard addition calibration was applied [44]. For cadmium (Cd) in urine, the samples were diluted 5-fold with 0.05 v/v Triton X-100 in 0.2% HNO_3_; 10 μL of the sample and 25 μg of (NH_4_)_2_HPO_4 _as a chemical modifier were injected into the THGA, pre-treated with 250 μg of zirconium and 20 μg of iridium [43]. Matrix-matched calibrations were applied for Pb, Se, and Cd. Total As was determined after transformation of the inorganic arsenic, As(III) + As(V), monomethylarsonate and dimethylarsinate, into similarly reactive species by treatment with L-cysteine. The final dilution of the urine samples was 5-fold. 10 mL aliquots were directly analyzed by hydride generation AAS [45].

We used the following reference materials for trace elements: Lyphochek Urine Metals Control Level 1, Cat. No. 400 (Bio-Rad Laboratories, Germany); Seronorm Trace Elements Urine Blank Cat. No. 201305; Seronorm Trace Elements Serum Level 1, Cat. No. 201405, and Level 2, Cat. No. 203105; Seronorm Trace Elements Whole Blood, Level 1, Cat. No. 201505, and Level 2, Cat. No. 201605 (Sero AS, Norway).

### Statistical analyses

Outcome variables were total protein, albumin, β_2_-microglobulin, creatinine in serum, CCR, kidney cortex width, and kidney length. To answer the question whether these outcomes are related to selenium serum concentration, lead in blood, and cadmium and arsenic urine excretion, we controlled for potentially confounding factors such as gender, age, history of smoking, diabetes mellitus, hypertension, body surface area (BSA), and creatinine in the morning urine sample, the 1-hour, and the 4-hour urine samples. We defined hypertension (Table 1) according to the The Seventh Report of the Joint National Committee on Prevention, Detection, Evaluation, and Treatment of High Blood Pressure [46]. For smoking, we distinguished between current smoker, ex-smoker, and non-smoker. We asked for presence of diabetes mellitus and determined hypertension in the first investigation (2003/04) and for new occurrence in the second investigation. BSA was used in the model to control for the effect of weight and height on kidney markers. In each investigation (2003/04 and 2004/05) the average kidney cortex width and kidney length of both kidneys were used as predictors.

**Table 1:** Characteristics of the Cohort of Adult Offspring of BEN Patients and Controls

		2003/04		2004/05	
		Offspring of BEN patients, n = 102 (%)	Offspring of control patients, n = 99 (%)	Offspring of BEN patients, n = 93 (%)	Offspring of control patients, n = 96 (%)
Gender	Men	50.0	47.5	48.8	47.9
Age	30–40	32.4	15.5	32.3	15.6
	41–55	43.1	57.6	43.0	58.3
	56 plus	24.5	27.3	24.7	26.0
Place of birth	BEN village	95.0	72.7	94.6	72.9
Smoking status	current smoker	39.2	34.4	38.7	35.4
	ex-smoker	22.6	12.1	21.5	12.5
	non-smoker	38.2	53.5	39.8	52.1
Diabetes		7.8	6.1	5.4	7.3
Hypertension in the offspring ξ		30.4	19.2	32.3	22.9
Other urinary tract diseases	kidney cancer	2.0	0	2.2	0
	kidney stones	10.8	4.0	10.8	6.5
	Pyelonephritis	10.8	0	9.7	1
	Hydronephrosis	1	0	1	0
	Cystitis	3.9	5.1	5.4	5.2
BSA ^# ^mean (standard deviation)	1.87 (0.22)	1.82 (0.21)	1.86 (0.18)	1.81 (0.17)	

ξ Systolic blood pressure ≥ 140 and/or diastolic ≥ 90 mm Hg [41]

# BSA = square root ((height in cm × weight in kg)/3600).

The intraclass correlation coefficient (ICC) is the between- subject minus the within- subject variance as a ratio to the sum of the two variances. A positive value of, for instance, 0.60 indicates that 60% of the variation is due to between-subject and 40% due to within-subject variance. ICC can become negative when the within-subject variance exceeds the between-subject variance.

In order to estimate the association between repeated measurements of arsenic, cadmium, lead, selenium, and kidney markers, we applied linear mixed models. Measurements of the two investigations are not independent and mixed models allowed for adjusting for within-participant effects [47]. The mixed model assumes that the random effects and the error vector are normally distributed, which was the case for kidney sizes and CCR; β2-microblobulin was log-transformed. SAS PROC MIXED was used to perform the regression analysis [48]. We used the Akaike information criteria and likelihood ratio tests to examine the significance of serial correlation (repeated statement) as well as to model random effects, along with a suitable variance-covariance matrix structure. To investigate whether offspring with a parental history of Balkan Endemic Nephropathy are more susceptible to metals and metalloids, we additionally stratified the models by parental history.

To compare the agreement between measurements of continuous variables in 2003/04 and 2004/05 we used the intra-class correlation coefficient (ICC). This is the between-subject minus the within-subject variance divided by the sum of the two variances. The ICC quantifies the proportion of total outcome variance that is due to inter-individual variation. A positive value, for instance, of 0.60 indicates that 60% of the variation is due to between-subject and 40% due to within-subject variance. ICC can become negative when the within-subject variance exceeds the between-subject variance. Due to methodological considerations [49], we chose not to directly adjust for creatinine using creatinine-adjusted parameters but to introduce creatinine as a confounder in the regression models.

## Results

Of the 201 participants from the first year, 189 participated in the follow-up (94%). Two were deceased, one moved out of the area, two could not be contacted, and seven decided not to participate further. Of the twelve lost nine were offspring of BEN patients. With the exception of hypertension, potential risk factors show similar distributions in each of the two investigations (Table 1). Of the BEN offspring, 30.4% had hypertension in 2003/04 and 29% in 2004/05. A lower prevalence was detected in control offspring (19.2 and 22.9%, respectively). BEN patients also reported more often a history of kidney stones and pyelonephritis.

Regarding arsenic and cadmium (measured in morning urine) and creatinine (measured in morning urine samples), we found no agreement between the two investigations for creatinine, a moderate agreement for arsenic and a good agreement for cadmium (Spearmen rank correlation and ICC, Table 2). For creatinine in morning urine samples the within-subject variance exceeds the between-subject variance. When we used the directly creatinine-adjusted parameters (division by the creatinine concentrations) the agreement for cadmium and arsenic was reduced, simply because of the high intra-individual variance of creatinine. There was a better agreement for two measurements, namely lead in blood and selenium in serum, measured one year apart.

**Table 2:** Metals and metalloids in urine and serum in two consecutive investigations

	Variable	N	Median	5%-value	95%-value	Spearman correlation coefficient (p)	ICC	ICC 5%-value
As in urine (μg/L)	2003/04	201	3.10	0.70	9.30	0.28 (0.0001)	-0.06	-0.18
	2004/05	189	2.90	0.80	8.90			
Cd in urine (μg/L)	2003/04	201	0.60	0.15	2.15	0.48 (< 0.0001)	0.44	0.34
	2004/05	189	0.70	0.27	1.91			
Creatinine in morning urine (g/L)	2003/04	201	0.72	0.15	1.80	-0.06 (0.40)	-0.47	-0.56
	2004/05	189	0.75	0.10	1.95			
Pb in blood (μg/L)	2003/04	201	90.90	47.10	213.00	0.70 (< 0.0001)	0.69	0.62
	2004/05	189	85.00	39.10	203.00			
Se in serum (μg/L)	2003/04	201	56.90	36.00	78.50	0.45 (< 0.0001)	0.17	0.05
	2004/05	189	72.10	46.40	95.70			

The intraclass correlation coefficient (ICC) is the between- subject minus the within- subject variance as a ratio to the sum of the two variances. A positive value of, for instance, 0.60 indicates that 60% of the variation is due to between-subject and 40% due to within-subject variance. ICC can become negative when the within-subject variance exceeds the between-subject variance.

Regarding the kidney parameters, Spearman rank correlation and ICC were low for total protein and albumin in serum, for creatinine in the 1-hour and 4-hour urine sample, and for the glomerular filtration rate (Table 3). These values did not improve when we directly adjusted for creatinine, since the within-subject variance of creatinine was high. The agreement between the sonographic determinations of kidney dimensions (kidney cortex and kidney length), β_2_-microglobulin, and the estimated creatinine clearance (CCR) was sufficient to conclude that they measure some underlying disease processes. In the following analyses we focus on these four parameters to test whether arsenic and metals explain their variance.

**Table 3:** Clinical markers of kidney function in two consecutive investigations

	Variable	N	Median	5%-value	95%-value	Spearman correlation coefficient (p)	ICC	ICC 5%-value
Total protein in urine (mg/L)	2003/04	201	30.0	10	140	-0.03 (p = 0.73)	0.03	-0.09
	2004/05	189	13.5	0	86			
Albumin in urine (mg/L)	2003/04	201	5.9	0.9	36	0.05 (0.53)	-0.14	-0.26
	2004/05	188	1.0	0.9	17			
β_2_-micro-globulin in urine (ng/mL)	2003/04	201	25.1	7.3	171	0.41 (< 0.0001)	-086	-0.89
	2004/05	189	37.5	12.5	244			
Creatinine in 1-hour urine (g/L)	2003/04	188	0.27	0.06	1.23	0.29 (p < 0.001)	0.23	0.11
	2004/05	189	0.44	0.11	1.7			
Creatinine in 4-hour urine (g/l)	2003/04	201	0.19	0.05	1.43	0.05 (0.47)	-0.04	-0.16
	2004/05	189	0.13	0.03	0.54			
Glomerular filtration rate/BSA^#^	2003/04	200	59.8	22.4	292	0.07 (0.33)	-0.16	-0.27
	2004/05	189	41.5	5.6	144			
CCR (Cockcroft & Gault)/BSA^# ^(mL/min/m^2^)	2003/04	199	58.2	41.6	79.7	0.264 (< 0.0003)	0.24	0.12
	2004/05	189	54.4	38.4	87.0			
Kidney length (average of both) (mm)	2003/04	201	118.5	106	128.5	0.91 (< 0.0001)	0.92	0.9
	2004/05	189	118	106.5	129.5			
Minimal kidney cortex width (average) (mm)	2003/04	201	16.0	12	18.5	0.68 (< 0.0001)	0.66	0.58
	2004/05	189	15.5	12	19			

# BSA = square root ((height in cm × weight in kg)/3600)

We did not find any significant effect of arsenic and metals on kidney cortex width (Table 4). Of the metals and metalloids analyzed, only selenium showed an association with kidney length, β_2_-microglobulin, and the estimated CCR in the models presented in Table 4. Lead gained importance only in the control offspring. Similar to selenium, it seems to decrease CCR.

**Table 4:** Association of metals and metalloids with β_2_-microglobulin, creatinine clearance, and kidney length controlling for confounders

	β2-microglobulin (ng/mL)			CCR Cockcroft, Gault/BSA (mL/min/m^2^)			Kidney length (mm)		
	Total sample	Ben-offspring	Control-offspring	Total sample	Ben-offspring	Control-offspring	Total sample	Ben-offspring	Control-offspring
	n = 201, obs = 387	n = 102, obs = 192	n = 99, obs = 195	n = 201, obs = 388	n = 102, obs = 193	n = 99, obs = 195	n = 201, obs = 388	n = 102, obs = 193	n = 99, obs = 195
Intercept	1.260	0.992	1.358	98.33	88.30	105.97	100.32	98.41	99.91
Blood lead (μg/L)	-0.001	-0.001	-0.001	-0.02	0.01	-0.06*	0.005	-0.01	0.00
Selenium in serum (μg/L)	0.001	0.003*	0.000	-0.09*	-0.05	-0.14*	0.02*	0.04*	0.01
Cadmium in urine (μg/L)	0.042	0.074	0.003	-2.09	-2.98	-1.53	0.10	0.11	0.05
Arsenic in urine (μg/L)	0.001	0.001	0.002	0.18	0.37	0.17	-0.06	-0.11	-0.07
Sex, male	0.189*	0.235*	0.151*	8.88*	4.70	13.09*	1.92	1.19	2.45
Age (years)	0.004*	0.008*	0.002	-0.67*	-0.57*	-0.69*	0.01	-0.02	0.02
Current smoker	-0.095*	-0.233*	-0.004	-0.30	2.89	-3.01	0.29	2.77*	-0.48
Ex-smoker	0.024	-0.059	0.039	-1.53	3.57	-8.33*	-0.12	0.98	-0.09
Diabetes mellitus	0.058	0.210	-0.063	-2.10	-2.99	-3.99	-1.87	-4.01	-0.25
Hypertension	0.048	-0.040	0.100	1.11	-0.11	2.42	-0.89	0.53	-1.72
Body surface area ^#^	-0.174	-0.139	-0.116	-	-	-	8.58*	8.74*	9.55*
Cystitis	0.014	0.032	-0.010	0.01	4.07	-1.41	-0.67	-1.07	-0.42
Pyelonephritis	-0.081	-0.118	0.191	2.39	2.33	-14.38	-0.65	0.77	-0.50
Kidney stone(s)	0.017	0.014	0.123	2.34	2.57	1.62	-0.57	-1.53	0.18
Creatinine in 1-hour urine	0.475*	0.417*	0.530*				-	-	-
Creatinine in morning urine (g/L)	0.018	0.004	0.006	-2.81*	-2.04	-3.57	-0.07	0.35	-0.28

*p ≤ 0.05

# BSA = square root ((height in cm × weight in kg)/3600)

Higher selenium levels are associated with a longer kidney length in the total group and the BEN offspring. When comparing an individual with 70 μg/L with one having 20 μg/L selenium, on the average kidney length would be increased by 1 mm (50 *0.02 mm). This is only a small increase compared to the total kidney length (approximately 120 mm). However, in BEN offspring the respective increase is 2 mm (50 *0.04 mm; Table 4). Selenium seems to decrease CCR in the total group and the control group, but not in BEN offspring. When comparing an individual with 70 μg/L with one having 20 μg/L serum selenium, CCR would be reduced by 4.5 mL/min/m^2 ^(50 *0.09 mL/min/m^2^) in the total group. This is a minimal decrease of CCR (range: 54–87 mL/min/m^2^). In the control group, lead is associated with a decrease (0.14 mL/min/m^2^). β_2_-microglobulin was log-transformed to achieve a normal distribution. A 50 μg/L increase in selenium would be approximately equivalent to a 1.4% increase in β_2_-microglobuline [10^(50*0.003)^].

## Discussion

Of elements investigated, cadmium and arsenic were associated neither with kidney dimension nor with function (hypotheses 3 and 4). Lead had a negligible effect on CCR (hypothesis 5). Selenium showed weak but significant associations with three of the four kidney parameters tested, namely kidney length, estimated CCR, and β_2_-microglobulin, but not kidney cortex width.

Our study included only BEN villages. Others think that the soil geochemistry in villages affected and not affected by BEN may be an etiologic factor [14]. It has been suggested that arsenic, cadmium, and lead (Pb) are elevated in the soil of BEN areas, whereas selenium is deficient [12,14,50]. Ecological studies do not take individual exposures into account. Nevertheless, according to these investigations, we would have expected higher levels of arsenic, cadmium, and lead in our participants. To examine whether the levels in our samples were different, we compared these to other reports on metal and metalloid exposures in Bulgaria. Tsolova *et al*., when studying copper-smelter workers, described urine arsenic concentration of 7.73μg/L in a control group [51]. We found approximately 3μg/L in an unexposed population (Table 2). Regarding cadmium, two Bulgarian investigations reported urine levels of 0.8–1.0μg/L [52,53]; we had urine concentrations of 0.6–0.7μg/L. However, adjusted for creatinine, urinary cadmium levels were higher in our Bulgarian samples (approximately 0.85μg/g creatinine) than levels reported for the United States (0.26μg/g creatinine) [54]. Lead blood concentration in our samples was lower (median of 90 μg/L in 2003/4, Table 3) than levels reported for occupational exposures in Bulgaria [53,55]. Selenium levels in this study (range 36–95 μg/L, medians: 56.9 and 72.1; Table 2) were comparable to levels reported in other studies in Bulgaria (57 and 66.5 μg/L) [30,56]. However, serum selenium levels seems to be lower in this rural region of Bulgaria compared to other countries [57,58]. For instance, in the United States, mean selenium levels were 125.7 μg/L. However, in rural regions selenium serum levels are lower; in central France on average 79 μg/L and in sample of rural Serbia on average 38.2 μg/L [57,59]. These concentrations are within the 95% confidence limit of our study (Table 2). In summary, concentrations of arsenic, cadmium, lead, and selenium in our samples were either comparable to concentrations in samples taken from other subpopulations in Bulgaria. Hence, these comparisons do not support the suggestion that BEN families are exposed to higher levels of arsenic, cadmium, selenium, or lead that could contribute to the occurrence of the disease.

Our study subjects were offspring of BEN patients who are likely to have a higher susceptibility of their kidneys to adverse exposures (hypothesis 5). Therefore, we expected to find that some of the elements would be associated with kidney function in BEN offspring. However, compared to controls, BEN offspring were not more affected (Table 4). For lead (Pb) we found a weak association with creatinine clearance, but only in control offspring. Further, since our investigation is based on offspring who have not yet developed the disease, we believe that this excluded behavioral changes such as avoidance of chemicals, which could have distorted the results. Therefore, since prior studies that reported associations between arsenic/metals and BEN had an ecological design or were based on geochemistry analyses, our negative findings suggest that non-epidemiologic studies may have been misleading.

A limitation of our investigation is the high intra-individual variance of kidney parameters. If our analysis would have been based on a single examination, this could have led to biased results. However, we included repeated measurements that took intra-individual variances into account and provided added confidence in our results. In addition, we focused on more stable indicators and did not consider clinical markers such as creatinine in urine, which showed a large variability over time. A second limitation is that offspring of BEN patients were younger (Table 1) and degenerative kidney disorders often occur in older people. Hence, it is possible, that we underestimated the effect of the metals/metalloids. Thirdly, as outlined in the introduction, there are no BEN patients born in non-BEN villages. Hence a two-way analysis (metals/metalloids and BEN-village) is not feasible. Since many new conclusions on what causes BEN have been presented based on aggregative (village data), we preferred a more rigorous testing of risk factors and focused on the association of metal/metalloids and BEN status within villages who are labeled BEN villages.

We are not aware of another study that investigated the association between kidney parameters and selenium in humans. Nevertheless, a recent study on selenium supplementation found an increased risk for type 2 diabetes [60]. Based on other findings, we expected that selenium would have a protective effect (hypothesis 1). However, we found three unexpected findings: Serum selenium was associated with increased β_2_-microglobulin, with increased kidney length, and decreased CCR. These may be chance findings. However, the US Agency for Toxic Substances and Disease Registry lists a number of comparable kidney effects in animals following exposure to selenium at levels several hundred times higher than normal human intake [38]. Effects include hydropic degeneration in sheep [61], increases in kidney weights in mice and ducks [62-64], larger Bowman's capsules and minimal papilla degeneration of the kidneys in rats [62,65], and glomerulonephritis and proximal convoluted tubule nephropathy in long-tailed macaques [66].

Recently, Balkan endemic nephropathy has attracted some attention [9-11]. First, a number of potential new risk factors have been presented. Second, its uniqueness in the Balkan regions has been questioned as similar manifestations were observed in other regions too [10], for instance in Sri Lanka [67]. There is a necessity for carefully testing various proposed risk factors and pathological mechanisms. We believe that our study contributes to the evidence that metals and metalloids are no risk factors for BEN. Further studies are necessary to test the claim of other proposed risk factors.

## Conclusion

Our results do not support the hypotheses that cadmium, lead, or arsenic pose a risk for the development of Balkan Endemic Nephropathy. Against the assumption in the literature, selenium was not protective, but a risk factor for all three markers: β2-microglobulin, creatinine clearance, and kidney length. Since comparable associations with anatomical and functional markers were observed in animals, future studies are warranted to investigate selenium effects on the kidney in humans.

## Abbreviations

arsenic (As), atomic absorption spectrometer (AAS), Balkan Endemic Nephropathy (BEN), body surface area (BSA), cadmium (Cd); creatinine clearance rate (CCR), electrodeless discharge lamps (EDL), intra-class correlation coefficient (ICC), lead (Pb), selenium (Se), transverse heated graphite atomizer (THGA)

## Competing interests

The author(s) declare that they have no competing interests.

## Authors' contributions

PD and WK designed the study, analyzed the data, and prepared the manuscript. VS and AB conducted the clinical investigations and helped with the preparation of the paper and the clinical interpretation. RG led the measurements of the metals and metalloids and reviewed the manuscript. ST provided toxicological expertise, help with the implementation of the study, data preparation, analyses, and the writing of the manuscript. All authors have read and approved the final manuscript.
